# Fight COVID Milwaukee protective behaviors and risk communications associated with the COVID-19 pandemic

**DOI:** 10.1038/s41598-023-49829-0

**Published:** 2023-12-22

**Authors:** Mohammad Titi, Aliyah Keval, Emma Martinez, Julia Dickson-Gomez, Staci Young, John Meurer

**Affiliations:** 1 Medical College of Wisconsin, 8701 W Watertown Plank Rd, Milwaukee, WI 53226 USA; 2 Georgetown University, 3700 O St NW, Washington, DC 20057 USA; 3 Institute for Health and Equity, Medical College of Wisconsin, 8701 W Watertown Plank Rd, Milwaukee, WI 53226 USA; 4 Department of Family and Community Medicine, Medical College of Wisconsin, 8701 Watertown Plank Rd, Milwaukee, WI 53226 USA

**Keywords:** Public health, Disease prevention

## Abstract

The COVID-19 pandemic has had a major impact on society, causing significant disruptions to everyday life. Risk communication strategies can play an important role in risk management as they allow individuals to prepare for and respond to public health emergencies appropriately. The aim of this study is to investigate public risk behaviors, perceptions of risk and risk communication, and experiences with COVID-19 to better understand the impact of COVID-19 on our community and to better inform public health decisions about communicating and reducing personal risk. Nine virtual focus groups were conducted with 79 residents of Milwaukee County. Audio transcripts of focus group recordings were qualitatively analyzed using MAXQDA. Predominant themes identified include public risk protective behaviors, the emotional toll associated with lockdown measures, and risk communication. Our findings provide a better understanding of how adults, African American and Hispanic groups in particular, viewed the risk communications and protective behaviors associated with COVID-19, how their lives were impacted by the pandemic, and how to effectively communicate public information about personal risk. These findings can help guide risk communication efforts and public health policy interventions for potential infection outbreaks in the future.

## Introduction

The SARS-CoV-2 (COVID) pandemic has had a profound global impact since its emergence in 2019, altering nearly all aspects of daily life. As of July 2023, the World Health Organization has reported that a total of 768 million cases have been confirmed globally, more than 6.9 million deaths have been reported, and 13 billion vaccine doses have been administered^1^. The United States continues to lead globally in confirmed COVID-19 cases and deaths, with over 100 million confirmed cases and over one million deaths^1^. During the peak of new cases in 2020, important public health safety measures were recommended or mandated to stop the viral spread at the federal and state levels. These measures included guidance on handwashing, respiratory etiquette, physical distancing, face coverings, quarantine, and eventually, a nationwide lockdown^2^.

The spread of the COVID pandemic further underscored health disparities present in the United States. Racial and ethnic minorities have been disproportionately impacted by the pandemic with increased risk for COVID infection, severe illness, and death^3–5^. These disparities have been driven, in part, by factors such as underlying health conditions, housing conditions, inadequate access to healthcare, occupational risk, and discrimination^6^. In addition, minority populations have higher rates of comorbid chronic conditions, further increasing their risk for severe illness and death^7,8^. Other groups, such as people of low socioeconomic status have also shared a disproportionately high burden of COVID^3,9^.

Risk communication can be an effective method of influencing public behaviors and reducing personal, household and community risk^10,11^. Public health officials can mitigate disease spread by disseminating information that enables at-risk individuals to make informed decisions and take protective measures. This information can be a key component of public health interventions, particularly during health emergencies, as a higher perception of risk has been shown to lead to more engagement in preventive behaviors and more compliance with public health recommendations^12,13^. As such, information about risks must be conveyed effectively to the public, and especially to vulnerable populations.

Fight COVID Milwaukee is a community-engaged research project that used COVID antibody test data, surveys, focus groups, and health records from adults living in Milwaukee County to analyze COVID risks from a population perspective. This paper examines the ways in which individuals responded to risk communication during the COVID pandemic by making lifestyle modifications to mitigate potential risks, as well as the experiences and significant emotional impact that resulted from these changes. Additionally, using information from focus group discussions with community members, Fight COVID Milwaukee provided a better understanding of how adults, and racial and ethnic minorities in particular, viewed the risks of COVID, and how to effectively communicate information about personal risk with the public. This information provides public health officials with knowledge to help guide and improve risk communication and public health policy.

## Methods

### Sampling and recruitment

Individuals enrolled in the Fight COVID Milwaukee (FCM) antibody and survey study were recruited to participate in the focus group study. Recruitment involved intentionally oversampling Black and Hispanic individuals, low-income, as well as elderly populations within Milwaukee County. Information letters and flyers about the focus groups were distributed through twenty primary care health centers, twelve community and faith partners, and three local health departments around Milwaukee, where these populations are prevalent. Interested individuals completed a screening survey. Eligibility criteria included being 18 years of age or older, speaking English or Spanish, and being able to provide informed consent. A total of 102 eligible participants were contacted and informed of the research purpose, focus group procedure, risks and benefits, and compensation. Informed written consent was obtained from all participants in their native language (English or Spanish) according to participants’ preference. The protocol was approved by the Medical College of Wisconsin Human Research Review Board and all methods were performed in accordance with the relevant guidelines and regulations.

Participants were placed into groups of 8–10 individuals based on similarities in age and race/ethnicity to extrapolate themes based on demographic similarities. One focus group was conducted in Spanish and the remaining 8 in English via Zoom for 90 min. Participant recruitment and focus groups were held in March and April 2022 during the Omicron surge.

### Focus group discussion content

Focus group interview questions centered around several topics, including feelings about medical research, understanding of COVID antibodies, behavioral changes in their lives due to COVID including social distancing, childcare, food security, housing, experiences with COVID illness, and trust in the COVID vaccine. Additionally, participants were asked to react to the Fight COVID Milwaukee web-based risk assessment tool presented by focus group facilitators to determine the most understandable and acceptable way of communicating community and individual risk^14^.

### Analysis

All focus groups were audio recorded and transcribed verbatim. Multiple researchers conducted analysis using the qualitative data analysis software MAXQDA. Codes were utilized to identify major topics of discussion among focus group participants. Initial codes were developed and refined collaboratively among the research team using an iterative process. Discrepancies in code labels were discussed in team meetings and a consensus was reached among members. The team then identified general themes representing data across all focus groups. A constant comparison approach was used to determine the salience of themes within and between groups, and by organizing themes into groups and subgroups. Analysis continued until no new significant themes emerged. Authors had access to participant demographic information during and after data collection in order to identify themes throughout the analysis process. Similarities and differences in themes were explored within and between groups of different demographic makeup.

## Results

Following their consent to participate in the study, participants completed a questionnaire noting COVID illness and vaccination, sociodemographic information, household and family size, employment, and pre-existing conditions. A total of 79 individuals participated in a focus group, representing a diverse range of age, gender, race/ethnicity, and social backgrounds (Table 1). Analysis of the Fight COVID Milwaukee focus groups identified three broad themes by community members regarding the COVID pandemic: public risk protective behaviors during the pandemic; emotional toll associated with lockdown measures; and risk communication. Each theme presented with substantive sub-themes are discussed below.

**Table 1:** Demographics of community FCM focus groups.

Characteristic	N (%)
Gender	
Male	24 (32%)
Female	47 (64%)
Other	3 (4%)
Age	
20–35	18 (24%)
36–50	22 (30%)
51–60	18 (24%)
65+	16 (22%)
Race/ethnicity	
Non-Hispanic White	27 (36%)
Hispanic White	21 (28%)
Black/African American	19 (26%)
Asian	4 (5%)
Other	3 (4%)

### Risk protective behaviors during the pandemic

A primary theme of our discussions revolved around adapting to the pandemic by engaging in protective behaviors to reduce personal and household risk. All participants reported high levels of self-adherence to recommended social distancing measures. Most participants’ risk protective behaviors included reducing exposure to the virus by social distancing, wearing facemasks, and frequently sanitizing. One participant anecdotally shared: “I’m just doing everything, sanitization, hand wash, face wash, whatever, a million times a day. As a matter of fact, my cuticles were so messed up for a while from so much hand washing” (African American female, 50 years of age—FG3). Many others, however, shared some variation of the following:

We stayed home. We did the bubble, we did limit all of our outings, a lot of masks, a lot of sanitizers (White female, 45 years of age—FG5).

Additional risk protective strategies included minimizing person to person contact in other aspects of everyday life. Some participants used online services more regularly, as described by one member: “I was a virtual churchgoer. I ordered curbside pickup at Sendik’s [a supermarket]. I ordered Amazon Fresh” (White female, 77 years of age—FG1); while other participants adopted completely new routines:

I made sure to go at nighttime when there was less traffic, masked up every time I went out of the house. (White gender non-conforming, 26 years of age—FG6)

We went grocery shopping once a month…my daughter did the grocery shopping and just put it on the porch. She’d stand on the sidewalk, and we would go out and pick it up off the porch. (White female, 75 years of age—FG1)

Participants across all focus groups discussed their different protective strategies aimed at reducing their risk of infection. Despite reported high levels of self-adherence to social distancing measures among focus group participants, many highlighted feelings of frustration towards others who did not share the same level of caution. Non-adherence was typically observed in younger populations, with the highest instances noted among participants who were obligated to be in specific settings, such as their essential workplaces. One participant stated: “Sometimes it’s really not about how safe you keep yourself. It really depends on other people as well. Because my boyfriend had gotten a job at a UPS warehouse facility and the people there were not taking any sort of COVID precautions. And as a result, he ended up catching it. I tested negative with the antibody test, but I did have all the symptoms” (White Female, 26 years of age—FG6). Other members shared similar experiences as well:

It was very frustrating after all of those months of taking those precautions to keep my family safe, that I was now in a very uncontrollable situation where I had to be in the same room with a lot of people who did not take mask wearing very seriously. (White female, 40 years of age—FG5)

But it’s difficult…in that not all people follow the precautions, and they practically don't care how close you are. So, yeah. But I think I did my part. (Asian female, 23 years of age—FG6)

Because I think a lot of people weren’t really adhering to the rules. I was personally also faced with a lot of individuals who did it on purpose…a lot of these incidents have kind of made it seem as though the implementation was a little lacking. (Mixed race female, 28 years of age—FG8)

Observed non-adherence to guidelines among younger populations was believed to be partly due to contradictory messaging at the start of the pandemic, as one participant stated Now, you want to draw the young people in to get vaccinated, when before they were superheroes. They couldn’t catch it. (African American female, 53 years of age—FG3). Many others also shared their thoughts on why younger individuals were less adherent to guidelines:

The teenagers, they don’t really take it seriously. I don’t know if it’s because of them, the news, and what they hear as far as teenagers’ immune system being somewhat resilient in having COVID…You know teenagers, and people think they are invincible, and I’m still seeing a lot of that. (Hispanic female, 42 years of age—FG9)

As a result, feelings of uncertainty around social reintegration were pervasive, and many felt held back by those who refused to adhere to public safety guidelines. Some even spoke on the need for herd immunity through the vaccine and being held back by others as well: They’re part of the problem as to why we have these variants. Until they get vaccinated, I feel like we’re just going to continue to have more variants pop up. (White female, 67 years of age—FG1) Nonetheless, participants acknowledged feeling more comfortable being in public once fully vaccinated; however, many still continued to practice risk protective behaviors:

I felt safe after I was fully vaccinated. My husband and I both waited the full two weeks…We will still always take a mask with us when we go out. (White female, 67 years of age—FG1)

So, I continue to take very seriously the whole masking, and to the greatest extent possible social distancing. I carry a bottle of hand sanitizer around my neck. I continue to just really, really, really limit my contact. (African American female, 43 years of age—FG9)

Overall, participants displayed high degrees of social consciousness and adherence to risk protective behaviors throughout the pandemic, perhaps due to openness to effective messaging. Regarding social reintegration, participants continued to practice protective behaviors given the increased spread of COVID variants and vaccine pushback. Many shared additional thoughts on long term social consequences as a result of the pandemic:

I think people are still hesitating shaking hands or hugging…I think we’ve kind of taken a step back a little bit from some of the usual behaviors we might have engaged in in the past. (White female, 80 years of age—FG1)

Prior to the pandemic, my children were comfortable with catching the bus. And now they outright refuse catching the bus. And when I try to make them get on the bus, they basically say, “It’s COVID on the bus.” (African American female, 44 years of age—FG2)

### Emotional toll associated with lockdown measures

Focus group participants reflected on the impact of social isolation during the pandemic and the emotional toll associated with lockdown measures. Across all focus groups, participants noted struggles as a result of restrictions on gatherings and social interactions. Older participants frequently highlighted their experiences with social isolation and loneliness, while younger participants predominantly discussed their challenges with remote learning and school disruptions. One participant shared: “It limited everything that I knew. Everything that was precious and valuable to me had to be cut out. I just had to ride the storm out. And I thank God for giving me the strength and the ability to do that, and to be able to deal with that” (African American male, 67 years of age—FG3). Others frequently used the word “difficult” when discussing their personal experiences during lockdown:

It’s been extremely difficult for people not to see one another physically (White female, 88 years of age—FG1).

It was a very difficult thing to go through because the people that I knew in my life, or know in my life, had been cut off. I couldn’t go to Illinois to visit my children or my grandchildren. The grandchildren that I have here, we kinda separated a little bit because we were unsure on what was going on with COVID…it just felt like, ‘My God, when is this ever gonna be over?’ (African American male, 64 years of age—FG3)

We are caregivers of my in-laws. It was quite difficult because they are very old and we had to stop visiting them, they were alone all the time, they were emotionally affected. (Mixed female, 47 years of age—FG7)

Many participants felt their lives were at a standstill because they were limited in their abilities to participate in their social life and regular daily activities with the restrictions. One participant stated: “It felt like somebody stole a year from us. It also feels like I don’t have that many good years left…It’s just horrible” (White female, 68 years of age—FG4). In addition, the transition to work from home and the transition to virtual learning appeared to amplify participant loneliness and feelings of isolation. One participant stated: “I missed that class interaction where I can just interject or ask questions. Also, I miss interaction with my peers” (Asian female, 23 years of age—FG6). Many shared similar experiences and overall negative feelings toward this transition:

Just the fact that kids were now being stuck in the house without their friends, I mean, just not having any social interaction that they are already stressed out, the parents are stressed out because of everything going on. The teachers are stressed because they have to learn how to navigate teaching online, so I think it was just a struggle for everyone. (White female, 38 years of age—FG5)

It was even worse when you onboard remotely and you’ve never met the people that you work with. You only have these screen-time relationships. It’s very difficult to develop rapport and that connectivity that you have from someone that you worked with in the office every day. (White male, 53 years of age—FG4)

In addition to the aforementioned feelings towards virtual interactions, participants were quick to share frustrations centered around the practical issues they faced when transitioning to a virtual setting. Many noted difficulties with student engagement to virtual learning: “They’re in the kitchen, and they’re supposed to be in class, and they’re supposed to be on their computer. And it was just a lot of work” (African American female, 52 years of age—FG3); while others reported significant learning loss as a result of the virtual learning environment: “My grandkids, they did virtual for a while. But it seemed like they had got behind. And now, since school opened up, they have to go to summer school to catch up because that virtual put them back” (African American male, 64 years of age—FG3). Participants across all focus groups had similar sentiments:

Virtual learning was terrible. My kids have always done well in person. And when they came home, it was such—every day, it was some kind of problem with me making them get online, making them stay engaged. It was just terrible every day. (African American female, 55 years of age—FG2)

So, he told me. He was like, ‘Nana, I don’t like this because it’s confusing to me. Because they’re telling me to do this and they’re telling me to do that. But when I’m struggling with something, I have to email back and forth. And it’s taking them too long to get back to me for the answers that I need in order to do my work properly.’ This is what I’m hearing from him. (African American female, 59 years of age—FG3)

So, like, we just had to use his tablet and stuff. But it’s hard because he’s 5, and he’s in K-5, and it’s hard to get him to sit there and use a tablet. You know, he doesn’t really get it much. And it’s really frustrating because he needs to be in school because, you know, he kind of just gets thrown off and stuff. It’s really frustrating. (Mixed race female, 27 years of age—FG8)

### Strategies communicating risk effectively

Effective communication of risk is essential to provide individuals with the information needed to guide their health behaviors, particularly in a pandemic. Many participants reflected on frustrations during the pandemic due to the ambiguity of information provided, especially during the beginning of the pandemic, that made guidelines hard to follow. Common criticisms included lack of clarity and lack of consistency in public health communication:

From the beginning, I think there was a lot of confusion about masks, and should you wear them and what should they be made of and whatnot else. And I don’t think that did any of us any favors. (White female, 75 years of age—FG1)

We got so conflicting advice about wearing or not wearing masks, what kind of mask you should wear, whether you should be four foot or six feet apart. So much conflicting advice. (White female, 80 years of age—FG1)

A recurring theme in conversations on communicating risk was the importance of honest and consistent information. One participant stated: “We really need the people that are giving us the information to be honest about the information. No matter what, be honest about it. Like I tell my grandchildren all the time, I can deal with the truth all day long. But when you [general] lie to me, that’s a problem”. (African American male, 64 years of age—FG3) Another participant added: “Consistency is important as well…I think an important group [younger individuals] got lost in the beginning, because of the way the information came out.” (African American female, 53 years of age—FG3) It was apparent throughout our discussions that participants felt the lack of consistency and lack of trust in government communication made it especially difficult to convince others to adhere to public safety guidelines.

To better inform decisions on communicating risk, participants were asked to reflect and provide their perspective on different strategies for effective risk communication. One participant spoke on the importance of clearly outlining risks and benefits, allowing individuals to make informed decisions (White gender non-conforming, 26 years of age—FG6). Another participant suggested using simple terms people can easily follow, sharing:

So, I was listening to Dr. Fauci. I listen to him quite often. And so, a lot of the information that he was giving out about the vaccination and all of that, it was really good information. However, he used so many acronyms. The ABB for the BBC. A lot of this information would throw a regular person off and they would be like, ‘What?’ (African American female, 52 years of age—FG3).

Minority participants, most notably African American individuals, discussed medical mistrust as a potential barrier to risk communication “I’m gonna speak as personally, I do have some major mistrust with our medical system just based on historically how Black folks have been treated or mistreated or not received treatment. So, that would make me very hesitant” (African American female, 52 years of age—FG2). Subsequently, they highlighted the importance of using “word of mouth” (African American female, 59 years of age—FG3) and using trusted community leaders to spread information to their local communities:

But when you’re able to find those key people that are like a catalyst in certain neighborhoods or certain networks and be able to disseminate that information in a thoughtful way, that isn’t the same. Having a very personalized script for people and being able to reach out to them, and then have somebody be able to have follow-up with resources, information. I think it goes a long way. (Mixed race male, 29 years of age—FG 8)

So, I think if you’re a trusted source, and you know you’re a trusted source of information, in your circle, or in your community, or whatever, you need to be straightforward with them and let them know. (African American female, 51 years of age—FG3)

Other strategies shared by members across all groups highlighted the importance of using personal experience, stating: “Personal experience brings it home more than anything else.” (White female, 77 years of age—FG1), and having more individualized risk communication. One participant shared: “I’m just thinking, just with risks, I do wish that we would’ve had a little bit more, I guess, tailored type of message. Because for different people there’s different risks, right? When you think about the 21-year-old versus the 65-year-old, that thought process and that risk benefit ratio looks very different” (African American female, 45 years of age—FG6). Finally, some participants touched on using social media presence to reach a wider range of audiences when disseminating information, especially when trying to influence younger populations:

When we are advertising them for parent-teacher conferences, we’re using those mediums, because we are able to, especially if you’ve got a Facebook presence or a social media presence, it’s really gonna look right to put your message out there. (African American female, 43 years of age—FG9)

## Discussion

Our focus group findings show unique and important ways in which racial and ethnic minority, low income, and elderly community members responded to the pandemic by adopting proposed public health recommendations, and the significant emotional impact that resulted from these changes. Considering many of these populations already assume the status of a marginalized group, it is essential to support these disadvantaged communities during public health crises such as COVID. We provide the important perspective of individuals on the government’s response to the COVID pandemic and how it impacted the community, as well as perspectives on effective communication strategies in the setting of a future pandemic. Using community-based focus groups, we were able to explore participants’ perception of risk and views on effective strategies for risk communication. The three main themes that were apparent throughout our focus group discussions pertaining to public perspectives on COVID were: public risk protective behaviors during the pandemic, the emotional toll associated with lockdown measures, and effective strategies for communicating risk during the pandemic. Differences in themes were explored within and between groups of different demographic makeup and discussed below. These themes explore public risk behaviors and experiences with COVID to better inform public health decisions about reducing personal, household, and community risk.

All participants reported high levels of self-adherence to safety guidelines and public health recommendations, commonly citing wearing a face mask, frequent sanitizing, and minimizing person to person contact. Despite their own adherence to guidelines, many participants also reported observing non-adherence in other community members, most notably in younger adults. Although this lack of adherence was mainly attributed to risk perception, other factors also played a significant role in shaping public attitudes towards COVID and public adherence to safety guidelines. For example, the rapid spread of misinformation and conspiracy theories about COVID led to confusion and mistrust of public health recommendations^15,16^. Additionally, exposure to COVID misinformation has been shown to be associated with lower adherence to public health recommendations including hand hygiene and physical distancing, and individuals who believe in COVID conspiracy theories are less likely to follow guidelines, such as wearing a face mask in public^17–20^. Furthermore, political affiliation has been shown to be a strong predictor of risk perceptions, policy preferences, and protective behaviors related to the COVID pandemic^21,22^. In some cases, public health recommendations have been framed as a political issue and have been contradicted by political figures, leading to resistance or noncompliance with measures such as mask-wearing and social distancing^23^. Overall, participants felt discouraged by public levels of adherence to safety measures and described feelings of uncertainty surrounding social reintegration. As a result, many continued to practice protective behaviors despite the relaxation of isolation measures and widespread vaccine availability.

In investigating the psychosocial impact of COVID and the experiences associated with public health isolation measures, we found that many participants shared similar experiences of psychological distress and negative feelings towards the pandemic lockdown period. Restrictions on social gatherings resulted in profound feelings of isolation, and the inability to gather with family members and loved ones seemed to have the greatest impact on emotional wellbeing. Although these experiences were widespread, we found that older participants expressed more feelings of loneliness and social isolation throughout our discussions. The COVID pandemic presented a unique and challenging situation in which many individuals and families experienced social isolation and loneliness for extended periods of time, and since older individuals were a population at high risk of serious infection, they faced the additional burden of heightened health concerns and anxiety throughout this difficult period. Younger participants, on the other hand, discussed how social isolation and feelings of loneliness were exacerbated by virtualization, and many noted that the social interactions felt unusual and unfulfilling in an online setting. In addition, frustrations and challenges associated with virtual learning, such as decreased student engagement and a perceived learning loss, added to overall negative feelings towards the lockdown period. These frustrations are supported by evidence showing that many students experienced some level of learning loss, with more significant learning loss in students of low socioeconomic status and minority populations^24^. These losses may translate to greater long-term challenges and health consequences in adulthood as education is one of the strongest predictors of socioeconomic status and health.

Finally, we sought to evaluate perceptions of risk communication and public perspectives on effective strategies for communicating risk. Our findings indicate participants found communication regarding COVID lacked clarity and consistency, especially at the start of the pandemic. Many noted that this inconsistency made efforts to convince others to adhere to safety measures especially difficult due to subsequent feelings of mistrust in government communications. Understandably, the rapidly changing nature of information about COVID and its transmission made it difficult for individuals, governments, and businesses to keep up. This dynamic situation contributed to confusion about the virus and its early messaging and allowed for easier spread of rumors and incorrect information online. Nonetheless, participants highlighted the importance of honesty and consistency from government sources when disseminating information regarding personal risk. Other points of emphasis in effective risk communication strategies included using personal experience, using layman’s terms, clearly outlining risks and having individualized risk communication, and utilizing social media platforms for wider outreach. Minority groups mentioned medical mistrust as a potential barrier to effective risk communication, and the importance of using local trusted community members to disseminate information in a manner that will resonate with minorities. Overall, participants felt implementation of these risk communication strategies would allow for greater public adherence in future settings; however, many acknowledged the limitations of risk communication in populations with high levels of mistrust, political polarization, and limited access to accurate information. Even though a variety of factors can influence public risk perception, risk communication interventions have been shown to improve risk understanding and directly lead to changes in public behavior^10,25^. Considering that not all risk communication approaches are effective, it is important to concentrate efforts on communication of risk-mitigation alternatives considered useful by the target audience. By understanding public perspectives on risk communication, public health officials can gain valuable insight that can be used to help guide and improve future communication, optimizing their reach to the target audience.

Limitations of this study included selection bias, as individuals participating in a focus group study were those who were more likely to be concerned about and adherent to recommendations. In addition, our small sample size relied on convenience sampling of community members from Milwaukee County. Consequently, the sample is balanced in age and race distribution, but the ratio of male to female is 1:2 in sex. During participant screening, emphasis was made on including vulnerable individuals from the urban community to gain insights into their perceptions of the pandemic. This selection process may have discouraged the participation of individuals with differing viewpoints, and the views of these participants may not represent the views of individuals in other communities, such as rural or suburban areas.

## Conclusion

The COVID pandemic had a profound impact on society and presented a considerable public health challenge to governments, public health, and health care systems around the world. Risk communication plays an important role in mitigating the spread of disease by informing individuals and allowing them to prepare for and respond to public health emergencies in an effective and timely manner. Our findings provide a better understanding of how socially vulnerable, urban residents from black and brown backgrounds viewed the risk and risk communications associated with COVID-19, how their lives were impacted by the pandemic, and how to effectively communicate public information about personal risk. This information can maximize the reach of public health officials reach, which can be a key component of public health interventions, particularly during health emergencies. This information can help guide risk communication efforts and public health policy interventions for other infection outbreaks in the future.

## Data Availability

The datasets and codes that support the results of this study are not publicly available because the participants did not provide consent for release of the data but are available from the corresponding author on reasonable request.

## References

[1] WHO. *WHO Coronavirus (COVID-19) Dashboard*. https://covid19.who.int/ (2023).

[2] Alexander M, Unruh L, Koval A, Belanger W (2022). United States response to the COVID-19 pandemic, January–November 2020. Health Econ. Policy Law.

[3] Wyatt Koma, S. A., Neuman, T., Claxton, G., Rae, M. & Josh Michaud, J. *Low-Income and Communities of Color at Higher Risk of Serious Illness if Infected with Coronavirus*. https://www.kff.org/coronavirus-covid-19/issue-brief/low-income-and-communities-of-color-at-higher-risk-of-serious-illness-if-infected-with-coronavirus/ (Kaiser Family Foundation, 2020).

[4] Holmes L (2020). Black-White risk differentials in COVID-19 (SARS-COV2) transmission, mortality and case fatality in the United States: Translational epidemiologic perspective and challenges. Int. J. Environ. Res. Public Health.

[5] Boserup B, McKenney M, Elkbuli A (2020). Disproportionate impact of COVID-19 pandemic on racial and ethnic minorities. Am. Surg..

[6] CDC. *What is Health Equity?*. https://www.cdc.gov/healthequity/whatis/index.html (2022).

[7] Tai DBG, Shah A, Doubeni CA, Sia IG, Wieland ML (2021). The disproportionate impact of COVID-19 on racial and ethnic minorities in the United States. Clin. Infect. Dis..

[8] Greenaway C (2020). COVID-19: Exposing and addressing health disparities among ethnic minorities and migrants. J. Travel Med..

[9] Dasgupta S (2020). Association between social vulnerability and a county's risk for becoming a COVID-19 hotspot—United States, June 1–July 25, 2020. Morb. Mortal Wkly. Rep..

[10] Winograd DM (2021). Rapid review of virus risk communication interventions: Directions for COVID-19. Patient Educ. Couns..

[11] Zhong W (2017). Simulating influenza pandemic dynamics with public risk communication and individual responsive behavior. Comput. Math. Organ. Theory.

[12] Cipolletta S, Andreghetti GR, Mioni G (2022). Risk perception towards COVID-19: A systematic review and qualitative synthesis. Int. J. Environ. Res. Public Health.

[13] Joslyn S (2021). COVID-19: Risk perception, risk communication, and behavioral intentions. J. Exp. Psychol. Appl..

[14] Keval A (2022). Community focus groups about a COVID-19 individual risk assessment tool: Access, understanding and usefulness. Res. Sq..

[15] Kouzy R (2020). Coronavirus goes viral: Quantifying the COVID-19 misinformation epidemic on twitter. Cureus.

[16] Pennycook G, McPhetres J, Zhang Y, Lu JG, Rand DG (2020). Fighting COVID-19 misinformation on social media: Experimental evidence for a scalable accuracy-nudge intervention. Psychol. Sci..

[17] Freeman D (2022). Coronavirus conspiracy beliefs, mistrust, and compliance with government guidelines in England. Psychol. Med..

[18] Romer D, Jamieson KH (2020). Conspiracy theories as barriers to controlling the spread of COVID-19 in the US. Soc. Sci. Med..

[19] Roozenbeek J (2020). Susceptibility to misinformation about COVID-19 around the world. R. Soc. Open Sci..

[20] Hornik R (2021). Association of COVID-19 misinformation with face mask wearing and social distancing in a nationally representative US sample. Health Commun..

[21] Allcott H (2020). Polarization and public health: Partisan differences in social distancing during the coronavirus pandemic. J. Public Econ..

[22] de Bruin WB, Saw HW, Goldman DP (2020). Political polarization in US residents' COVID-19 risk perceptions, policy preferences, and protective behaviors. J. Risk Uncertain..

[23] Stroebe W (2021). Politicization of COVID-19 health-protective behaviors in the United States: Longitudinal and cross-national evidence. PLoS ONE.

[24] Donnelly R, Patrinos HA (2022). Learning loss during Covid-19: An early systematic review. Prospects.

[25] Heydari ST (2021). The effect of risk communication on preventive and protective behaviours during the COVID-19 outbreak: Mediating role of risk perception. BMC Public Health.

